# Mechanisms Maintaining Mitochondrial DNA Polymorphisms: The Role of Mito-Nuclear Interactions, Sex-Specific Selection, and Genotype-by-Environment Interactions in *Drosophila subobscura*

**DOI:** 10.3390/insects16040415

**Published:** 2025-04-15

**Authors:** Pavle Erić, Marija Savić Veselinović, Aleksandra Patenković, Marija Tanasković, Bojan Kenig, Katarina Erić, Boris Inđić, Stefan Stanovčić, Mihailo Jelić

**Affiliations:** 1 Department of Genetics of Populations and Ecogenotoxicology, Institute for Biological Research “Siniša Stanković”—National Institute of the Republic of Serbia, University of Belgrade, Bulevar Despota Stefana 142, 11060 Belgrade, Serbia; aleksandra@ibiss.bg.ac.rs (A.P.); marija.tanaskovic@ibiss.bg.ac.rs (M.T.); katarina.eric@ibiss.bg.ac.rs (K.E.); 2 Faculty of Biology, University of Belgrade, Studentski trg 16, 11000 Belgrade, Serbia; marijas@bio.bg.ac.rs (M.S.V.); mihailoj@bio.bg.ac.rs (M.J.); 3 The Center for Promotion of Science, Kralja Petra 46, 11000 Belgrade, Serbia; bkenig@cpn.rs; 4 Forest Microbial Genomics Group, Faculty of Forestry, Institute of Forest and Natural Resource Management, University of Sopron, Bajcsy-Zsilinszky str. 4, H-9400 Sopron, Hungary; boris.indjic@phd.uni-sopron.hu; 5 Institute of Molecular Genetics and Genetic Engineering, Vojvode Stepe 444a, 11000 Belgrade, Serbia; stefan.stanovcic@imgge.bg.ac.rs

**Keywords:** mtDNA, mito-nuclear interactions, genetic diversity, sex-specific selection, genotype-environment interactions, life-history, intra-population variation, *Drosophila subobscura*

## Abstract

Experimental mito-nuclear introgression lines (MNILs) were established by backcrossing isofemale lines of *D. subobscura* originating from the same populations. MNILs were subjected to a series of life-history experiments designed to test the fitness of the bearers of different combinations of two main mtDNA haplotypes on their own nuclear background, as well as on the background of the opposite haplotype. By having 11 replicas of the four mito-nuclear combinations, we could test not only the adaptive significance of the differences between the two main haplotypes but also the influence of additional variation present within each of the 11 combinations on fitness. Testing the fitness of individuals of both sexes enabled us to examine if sex-specific selection has a role in maintaining the frequencies of the two mtDNA haplotypes in nature. Conducting the fitness assays on two different temperatures enabled us to test whether different temperatures favor specific mtDNA haplotypes or mito-nuclear genotypes and consequently promote stable sympatric mtDNA variation. The results show weak signature of genotype-by-environment interactions, and no sex-specific selection regarding differences between the two main haplotypes. However, individual models across different life-history components showed these two mechanisms at play in promoting mtDNA variability present in specific mito-nuclear crosses. Our models show that mito-nuclear interactions are, in fact, more important as units of selection.

## Simple Summary

*Drosophila subobscura* is an interesting model to study forces that shape and maintain sympatric mitochondrial DNA (mtDNA) variation, due to the widespread presence of the two main, almost equally frequent haplotypes. Experimental setups using different life-history components enable us to study the adaptive significance of mtDNA variation and its effects on fitness while also trying to disentangle the role of different balancing selection mechanisms that operate in order to promote stable variation in natural populations. Constructing mito-nuclear experimental lines using backcrossing enables us to discern whether mito-nuclear interactions play a role in maintaining the aforementioned mtDNA variation. Since studies examining the maintenance of intrapopulation mitochondrial variability are scarce, our experimental results significantly contribute to this field of research.

## 1. Introduction

The mitochondrial genome is circular, non-recombining [1], haploid, and uniparentally transmitted, and there is an absence of dominance. Since mtDNA is haploid, there are no heterozygotes, resulting in all alleles that influence differences in adaptive value being exposed to selection. Due to these characteristics, there was a long-standing consensus in the scientific community that the variability observed in mtDNA sequences resulted from accumulation under the neutral equilibrium model [2,3,4]. Although it has long been known that the variability present in mtDNA is not neutral [5,6,7,8,9,10,11,12] and is not simply a consequence of the high mutation rate in the mitochondrial genome [13], the mechanisms that maintain intrapopulation mtDNA variability have been hard to explain in the absence of overdominance [14]. For decades, balancing selection has been a key evolutionary concept in explaining the maintenance of adaptive genetic variability in general [15,16]. Mitochondrial genes are crucial for the efficient functioning of cells, given their role in the metabolism of eukaryotic cells, primarily in ATP production, which is impossible without the interaction of mitochondrially encoded proteins with nuclear-encoded ones. Therefore, one of the first proposed mechanisms for maintaining mtDNA variability involves mito-nuclear interactions [17,18]. The authors of both studies theoretically concluded that for stable mtDNA variation to be maintained through mito-nuclear interactions, additional conditions are necessary because of the unique characteristics of mtDNA. Gregorius and Ross [18] proposed a negative frequency-dependent selection (NFDS) model, which maintains variability in cytoplasmic and nuclear genes whose products are functionally linked. In this type of balancing selection, the fitness of a particular allele is inversely proportional to the frequency of that allele in the population. This mechanism’s role in the maintenance of a stable mtDNA polymorphism was recently demonstrated in natural populations of a seed beetle [19] as well as in *Drosophila subobscura* [20]. Another proposed mechanism is the action of sex-specific selection (SSS) on mito-nuclear genotypes. The theory [21,22] predicts that certain mito-nuclear combinations result in better fitness for individuals of one sex, while other combinations are favored in individuals of the other sex. In the two mentioned studies, the authors defined the conditions and, through theoretical considerations and numerical simulations, demonstrated that differences in fitness between the sexes, whether in the juvenile or adult stage, can maintain stable mito-nuclear polymorphisms. In addition to the mentioned balancing selection mechanisms, there is evidence that complex interaction between variable environment and mito-nuclear genotype [23,24] can help maintain variation in mtDNA, as well. According to this model, an individual of a specific genotype in a population facing variable environmental conditions will have a relative fitness that depends on environmental factors. These factors can vary spatially or temporally, with opposing mito-nuclear combinations providing an advantage at the extremes of the distribution of environmental variation.

*D. subobscura* is an exceptional model system for studying the mechanisms that shape and maintain intrapopulation mtDNA variability [20,25,26,27,28] because of the presence of two equally frequent haplotypes in nature. The analysis of *Drosophila subobscura* mtDNA variation started with restriction site analysis (RSA) of the entire mtDNA almost four decades ago [29]. The research that followed [30,31,32,33,34,35] all confirmed the widespread presence of the two main, almost equally frequent haplotypes, named I and II, and rare, population-specific haplotypes. Across the species’ range, with the exception of the Canary Islands, where an endemic haplotype (VIII) is dominant [36], these two haplotypes prevail with a combined frequency of over 90%.

Concerning RSA analysis, the main haplotypes differ in only one site specific to the *Hae*III enzyme. This SNP is located in the *ND5* gene [30,31] and is a synonymous substitution [37]. Sequencing of another gene (*Cyt b*) [38] did not reveal any consistent differences between the two haplotypes. A dominant haplotype and a large number of derived haplotypes, recorded only once, were observed. Another study sequenced the entire mtDNA of individuals from both haplotypes [20] and revealed another obligatory nucleotide difference between the two most common mtDNA haplotypes. This difference is not in a protein-coding sequence but in the 12S rRNA gene, and the differences in its sequence could potentially affect the secondary structure of rRNA. Since mitochondrial-encoded rRNA must structurally align with a complex of nuclear-encoded proteins to fulfill its role in protein synthesis, the differences between the two haplotypes in this species represent a promising model for studying mito-nuclear coadaptations.

Experimental analyses involving two mtDNA haplotypes have shown varied results. One study [32] found that under laboratory conditions, haplotype II became fixed in all population cages, regardless of the initial frequency of the two haplotypes. On the other hand, when mtDNA haplotypes I and VIII were placed in competition on different nuclear backgrounds in population cages, the haplotype on its own nuclear background always prevailed, suggesting an effect of mito-nuclear interactions [39]. Fitness component analysis experiments have yielded ambiguous results, with some fitness components indicating an adaptive advantage for haplotype I under laboratory conditions [40], while other experiments suggested an adaptive advantage for haplotype II in the laboratory environment [41]. These experiments, however, measured the fitness components of the two haplotypes on their own nuclear backgrounds. A comparison of the fitness of several mtDNA haplotypes on a uniform nuclear background (derived from a single rare haplotype) did not show differences in fitness components between the two haplotypes [42]. The fitness values of haplotypes I and II were not significantly different from each other, while different rare haplotypes were significantly worse or better compared with the two main haplotypes. These results suggest that the maintenance of sympatric mtDNA variability in this species is primarily influenced by selection acting on mito-nuclear coadaptations.

Previous research highlights the complexity of the mechanisms maintaining intrapopulation mtDNA variability in *D. subobscura*, implicating both neutral processes, namely periodic contractions and expansions of populations, and selection. Neutrality tests and the distribution of expected and observed nucleotide differences point to stochastic mechanisms, primarily seasonal bottlenecks followed by rapid population expansions, as well as a large population expansion after the last ice age, as the main causes of mtDNA variability patterns [25,38,43]. In optimal conditions, populations are rapidly increasing in number, which generates a multitude of rare variants from the two main haplotypes, while nucleotide diversity remains relatively stable [25,38]. However, the balanced presence of the two main haplotypes throughout the species range, and over time is intriguing and strongly suggests the action of some form of balancing selection. It is interesting to note that in natural populations, the two haplotypes change in frequency seasonally; more specifically, individuals with haplotype I are the most frequent in June, while they are outnumbered by those with haplotype II in other periods of the year [43,44]. So, different environmental conditions may be optimal for the two haplotypes. An interesting notion is also the linkage disequilibrium between chromosomal arrangements and mtDNA haplotypes that is sporadically found in natural populations [28,34,35,45] and gives the possibility that selection acts on joint mito-nuclear genotypes.

In this paper, we use a set of mito-nuclear introgression lines (MNILs), making it possible to separate the effects of mtDNA, nuclear background, and their interaction. We conduct fitness assays to test whether SSS operates to maintain the equilibrium of the two main haplotypes. We also test whether mitochondrial or mito-nuclear combinations show different fitness values in different temperatures, so we test the possibility that environmental variation supports stable variation in mtDNA in *Drosophila subobscura*. We focus on the fitness differences between the two most frequent haplotypes. However, since we used 11 independent replicas of the two haplotypes, which potentially harbor additional differences, our results have a broader impact on the effect of mtDNA variation on fitness and its maintenance.

## 2. Materials and Methods

### 2.1. Maintenance and Establishment of MNILs

The lines used in this study are a subset of isofemale lines (IFLs) formed from collected individual females in June 2015 at the foothills of Stara Planina, Eastern Serbia [38]. The two collecting sites were approximately three kilometers apart: Stara Kalna (SK) and Gabrovnica (G). These lines were previously genotyped using the restriction fragment length polymorphism (RFLP) and sequencing of the *Cyt b* gene. The presence of endosymbiotic *Wolbachia* in the IFLs was excluded using a PCR test because it is known that these endosymbionts can influence their host fitness [46,47]. In line with the aims of this study, lines possessing one of the two most frequent mtDNA haplotypes and the most common *Cyt b* haplotype were selected for experiments. In this way, taking into account the resolution of the methods, differences that may arise in rare, endemic haplotypes were partially excluded, and the experiments were focused on measuring phenotypic differences only between mitochondrial haplotypes I and II. Eleven lines of haplotype I were selected, and each was randomly paired with one line of haplotype II to form mito-nuclear introgression lines (MMILs) by backcrossing. It is worth mentioning that even though the two localities are very close to each other, an IFL from one locality was always paired with an IFL from the same locality, in order to have comparisons of sympatric mtDNA haplotypes.

During each generation of backcrossing prior to the life-history component experiments, vials containing pupal-stage individuals were kept in darkness, as newly hatched *D. subobscura* individuals will not mate in such conditions [48,49]. Each morning, the sexes of the hatched individuals from these experimental lines were separated using ether anesthesia. For each generation of backcrossing, ten virgin females of a specific mitochondrial haplotype were mated with 20 virgin males of a specific nuclear background.

**Figure 1:**
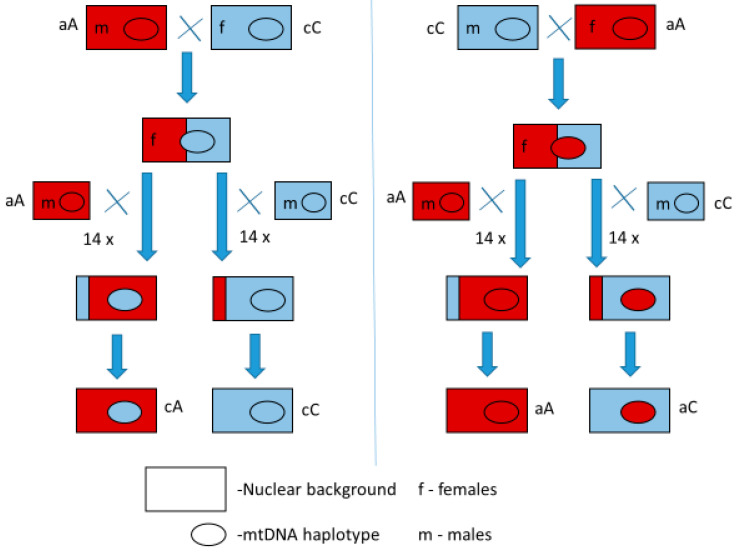
Scheme for the backcrossing procedure used for creating experimental MNILs. Red and blue colors indicate haplotypes a and c as well as their respective nuclear backgrounds.

Since mitochondria and their genomes exhibit exclusively maternal inheritance, females with a specific mitochondrial haplotype were mated with males of the desired nuclear background. By crossing females from the selected experimental lines for a minimum of 14 consecutive generations with males from the same IFL, the mitochondrial haplotype of the female was retained, while its nuclear background was gradually replaced with that of the IFL of the male over 14 generations. After this procedure, the proportion of the original nuclear genome of the female’s line would be approximately 0.006%, with each subsequent backcrossing generation further decreasing the proportion of the female’s original nuclear genome. The design of the backcrossing used to construct the experimental MNILs is shown in Figure 1. Through backcrossing, four mito-nuclear lines were created for each pair of IFLs, with all combinations of nuclear backgrounds and mitochondrial haplotypes (quartet). In this way, a total of 44 experimental MNILs (11 quartets) were obtained, whose fitness was compared through a series of experiments. After 14 generations of backcrossing, these MNILs were re-tested with RFLP analysis using *Hae*III restriction enzyme to exclude the possibility of working with lines of the wrong genotype before the fitness component experiments.

For clarity, haplotypes I and II were renamed using lowercase letters a and c, respectively, following the nomenclature of restriction patterns obtained using the *Hae*III enzyme. Nuclear backgrounds were labeled with uppercase letters A and C to avoid confusion. Within each of the eleven comparisons, there were four mito-nuclear combinations of haplotypes: aA, aC, cA, and cC. This design allows for the comparison of fitness components between the two most common mtDNA haplotypes, both on their own nuclear background and on the nuclear background of the reciprocal haplotype, across 11 independent groups or quartets. Additionally, this design enables the determination of the effect of each variable and their interactions on fitness, both within each of the 11 quartets and comprehensively across the entire dataset.

### 2.2. Fitness Measurements

The first fitness experiment measured resistance to desiccation, or the number of hours that individuals could survive in dry conditions without food and water. The experiment was performed at two experimental temperatures, 19 °C and 24 °C, while the air humidity in both groups was set to 30%. Two temperatures were chosen because haplotype I is more frequent during late spring, while haplotype II is more frequent during autumn, as well as the assumption that the reason for this is the adaptation of the two haplotypes to different temperature optima [43]. During the setup of the experiment, 20 males and females were collected from each experimental MNIL for each experimental temperature. The flies used in the experiment were virgin and between five and seven days old to eliminate the influence of reproduction and age on survival variability under dry conditions. Individuals were placed into small modular plastic tubes, where each tube was sealed with the next. Two 0.5 mm air holes were made in each tube using a needle. The tubes were stacked in columns of 20 tubes with flies for easier monitoring. Individuals who died within the first two hours were excluded from the experiment, as it was assumed that their deaths were due to injuries sustained during handling rather than dry conditions. After setting up the experiment, the flies were monitored hourly until the last individual had died. A fly was considered dead when it could no longer cling to the tube walls with its extremities or remain upright after rotating and gently shaking the tube column. Due to the fact that some individuals did not survive the transfer process into the experimental tubes, an average of 17.75 females and 16.88 males from each group per temperature were monitored in the experiment. With a total of 11 quartets or groups, each with four MNILs, at two temperatures, two sexes, the desiccation experiment was conducted on around 3000 *D. subobscura* individuals.

Following the sixteenth generation of backcrossing, a second major fitness analysis experiment was conducted by measuring three life history components: development time, viability, and percentage of males among all emerged adults. Development time represents the number of days required for an individual to develop from an egg to an adult. Viability represents the proportion of individuals that survive from one developmental stage to another. In this experiment, we measured survival rates from egg to pupa (EtP), from pupa to adult (PtA), and most importantly, survival of the entire development from egg to adult (EtA). The percentage of males essentially analyzes the sex ratio among all emerged adults and allows for the examination of sex-specific effects on juvenile fitness. Deviations from an equal sex ratio indicate differential survival of individuals of different sexes. In this part of the experiment, the development times from the egg through the pupal to the adult stage were measured across different experimental MNILs. Additionally, survival in each individual vial was calculated by counting the individuals that reached each developmental stage. In this experiment, the survival of laid eggs per vial was measured, both to the pupal stage and to adulthood. The percentage of males among the individuals that reached adulthood was also observed to analyze sex-specific survival during the juvenile stage.

For each experimental line, 40 virgin females and 60 virgin males aged 5–7 days were collected. The isolated flies were placed in 330 mL jars with standard cornmeal medium to mate for five days. After five days, the flies were transferred to a new empty jar, which had a Petri dish as a lid instead of a cotton plug. The Petri dishes were previously filled with standard cornmeal medium coated with a liquid yeast solution and then attached to the jar neck using adhesive tape. The new jars were inverted to stand on the Petri dishes with the medium where the flies would lay eggs. Each morning during the experiment setup, the old Petri dishes were removed and replaced with new ones. Eggs collected from the Petri dishes each morning were transferred under a binocular microscope to experimental vials containing cornmeal medium and yeast for development. A maximum of 15 vials per line was set up daily. To measure survival, 24 replicates, each with 24 eggs, were created for each of the 44 experimental lines at both experimental temperatures. The chambers where the vials with experimental eggs were kept were set to 19 °C and 24 °C. The light regimes accompanying these two experimental temperatures were designed to simulate different seasons. At 19 °C, there were 12 h of light and 12 h of darkness, while at 24 °C, there were 16 h of light and eight hours of darkness to mimic daytime length differences between seasons (autumn and spring versus summer). The reason for doing this was because the frequencies of the two most common haplotypes of *D. subobscura* oscillate during the year in natural populations. The experimental vials with laid eggs were rotated within the chambers every two days to ensure as homogeneous a temperature and as uniform conditions as possible for all vials during the 36-day experiment. For the experiment tracking development dynamics, one-third (8 out of 24 replicates from each group) of the vials set up in this experiment were randomly selected. These vials were checked daily during the experiment in climate-controlled rooms set to the experimental temperatures to ensure temperature differences did not affect development time. All emerged flies were counted, and their sex was determined under a binocular microscope after ether anesthesia. Additionally, the number of pupae was recorded daily in each vial. The remaining two-thirds of the replicates, which were not monitored in the development dynamics experiment, were checked after 36 days from setup, and within each vial, the number of pupae and adults was counted, and the percentage of males was calculated. For this experiment, 44 experimental lines were constructed at two experimental temperatures, with 24 vials each containing 24 eggs, totaling over 50,000 eggs set up.

### 2.3. Statistical Analysis

All results from the fitness experiments were statistically analyzed using R v.4.1.0 [50]. All figures were made in R using the ggplot2 package [51].

Data from the desiccation resistance experiments were analyzed using the Cox proportional hazards model [52], implemented in the survival package v.3.2-13 [53]. These data were not censored, meaning that the event being monitored was recorded for every individual who participated in the experiment as the experiments continued until the last individual died. There were four fixed factors in the models: mitochondrial haplotype (MT), nuclear background (NU), sex, and temperature (T). It is important to note that the temperature factor (T) throughout the text also includes the light regime associated with it. The full model, which included these four factors along with all first-, second-, and third-order interactions, was found to be the most appropriate in most cases. To ensure comparability of data across different quartets, all datasets (11 quartets and overall) were modeled with all interactions of these four factors. One challenge with Cox models is that if one of the factors does not have hazards proportional to the other factors, the model must be stratified according to that factor, resulting in the loss of information about the factor itself. In several models, there were comparisons where the assumption of proportional hazards was not met, so the model was stratified by the factor whose hazard function was not proportional to the other factors. This typically occurs when one factor has a far greater influence on the independent variable than the others, to the extent that the other factors may appear negligible in comparison. In such cases, the dataset must be divided into as many models as the stratifying variable has levels. In this study, that number was always two, as all factors had two levels each. This procedure results in two models, one for each value of the variable by which the model is stratified, and then the average effects of the other variables on the independent variable are calculated across these two models while the effect of the stratified variable itself could not be measured. This occurred in different models for different factors, so in some cases, information about the significance of these factors is missing. The assumption of proportional hazards was checked using the cox.zph function [53], and factors whose hazard function was not proportional to the other factors in the model were stratified. Each quartet was modeled separately, resulting in 11 replicates of comparisons of the same mito-nuclear combinations (11 models). There was also a composite model where data from all 11 quartets were analyzed together. In addition to the four fixed factors, this overall model included a random effect—the quartet. The quartet, representing membership in one of the 11 groups comparing haplotypes I and II, was treated as a frailty term, a method for defining random effects in Cox models. This approach accounts for the heterogeneity of unmeasurable covariates in statistical modeling. Since Cox models assume a homogeneous experimental population, introducing frailty defines which samples are not mutually independent and groups them together in a way. The *p*-values for fixed effects in the composite model were obtained using the joint tests functionality within the emmeans package v.1.7.2 because of the presence of frailty terms and stratified variables [54].

The development time was analyzed using mixed linear models implemented via the lmer function in the lme4 package v.1.1-25 [55]. For this component, full models were also used, incorporating all interactions between the four fixed factors (MT, NU, sex, and T), as well as the replicate number as a random effect. This random effect term was used because environmental differences between individual vials could affect development duration, meaning that individuals developing within the same vial were expected to be more similar to each other than to those from other vials. The models for this component used reduced maximum likelihood (REML) estimation and Type III ANOVA (Type III sum of squares) for group comparisons. The *p*-values for the ANOVA tests were obtained using the LmerTest package [56]. There were 11 classical linear mixed models for each of the 11 quartets, as well as a composite model that included all data. In this overall model, there was a complex random term where the replicate number of the vial was nested within the quartet. In the 7th quartet, the genotype cA resulted in only one male emerging in eight replicates, with no females. This caused issues in modelling this quartet, as the model had no data for the development time of cA genotype females at 24 °C. Therefore, for this component, there are only ten models. Although the 7th quartet was incomplete, the overall model included data from all 11 quartets since no group in the entire dataset was without entries.

Viability per vial was analyzed using generalized linear models (GLM) with the glm function in base R [50]. In each group, EtP, PtA, and Eta survival was recorded. The three basic factors in these models were MT, NU, and T. For all three components (EtP, EtA, PtA), a binomial error distribution was used, while the number of eggs or pupae, depending on the component, was used as the denominator. As with the previous components, there were 11 models in which the quartets were analyzed individually, along with a composite model encompassing all 11 quartets. The overall model, which was a generalized linear mixed model (GLMM), was created using the glmer function from the lme4 package [55]. In this composite model, quartet membership was defined as a random effect. Since the ANOVA function is not compatible with glmer, *p*-values were obtained using the joint_tests function from the emmeans package [54]. Since most individuals that survived to the pupal stage also completed their development to adulthood, the EtP survival results were very similar to the EtA survival values, while the PtA values were less informative, given that about 95% of individuals that pupated also reached the adult stage. For this reason, survival from egg to adult, in addition to encompassing the entire development of the flies, was the most informative component, so only the models analyzing this component will be presented, while the other two will not be discussed in detail. The results of the ANOVA for the models of survival from egg to pupa (EtP) and pupa to adult (PtA) are presented in additional Tables S1–S4 and Figures S1 and S2 in Supplementary Materials.

The percentage of males among the total number of emerged adults per vial was calculated to analyze the sex ratio. It was modeled using the same generalized linear models as viability, where 11 quartet models and a composite model were constructed. The same error distribution was used, while the total number of emerged individuals per vial was used as the denominator in the calculations. As with survival, quartet data were modeled using the glm function while the glmer function was used for the composite model, where belonging to a quartet was defined as a random effect, and *p*-values were obtained using the joint_tests function from the emmeans package [54].

## 3. Results

**Figure 2:**
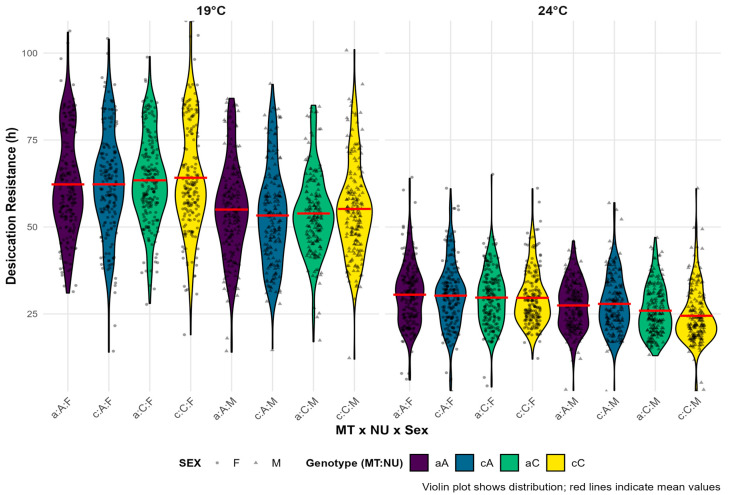
Violin plots showing the distribution of desiccation resistance for all combinations of genotypes and sex across all 11 quartets combined on two experimental temperatures. Individual data points are overlaid as jittered dots, with shapes distinguishing between sexes. The mean resistance time for each group is indicated with a red horizontal line. Colors indicate genotype combinations. The lowercase letter represents the mtDNA haplotype (MT), where “a” corresponds to haplotype I, while “c” corresponds to haplotype II. The uppercase letters represent the corresponding nuclear background (NU), while F and M denote females and males (sex), respectively.

The mean survival times under desiccation conditions for *D. subobscura* individuals from the composite model, encompassing data from all 11 quartets, are presented in Figure 2. The most noticeable observation from these graphs is the difference in survival between the two experimental temperatures, as individuals at the lower temperature survived, on average, about 70% longer than those at the higher experimental temperature. Additionally, it was observed that females survived approximately six hours (14%) longer on average (*p* < 0.0001). Looking at the entire dataset, a slight trend favoring nuclear background C at the lower temperature and nuclear background A at the higher temperature can be seen, as also shown in Table 1, where the interaction NU:T is statistically significant in the overall model. Regarding mito-nuclear combinations, no significant differences can be observed when considering the entire dataset, although different combinations of genotypes were favored in different quartets. The experiment did not show a preference for a specific mitochondrial haplotype in either temperature regime, as seen from the similar survival times of the haplotypes. When examining individual quartets, it can be noted that in some, MTa appeared better adapted to drought conditions, while in others, MTc showed greater resistance.

The results of the ANOVA for the overall model of survival time under desiccation stress in *D. subobscura* are presented in Table 1. The overall model had to be stratified by temperature because it violated the proportional hazards assumption. In the absence of temperature, sex had the greatest influence, followed by the interaction between NU:T and the nuclear background (NU) itself. In the composite model, the following interactions were also statistically significant: NU:sex, sex:T, and MT:NU:T. The results of the ANOVA for the 11 quartet models for survival times under desiccation stress in *D. subobscura* are presented in Table 2. The results show that temperature is the most significant factor in determining survival time under drought conditions. Four models had to be stratified by temperature because their hazard functions did not meet the proportionality assumption with the other factors, while in the remaining seven models, temperature was the most influential factor, with the highest statistical significance. In addition to temperature, sex emerged as the second most influential factor, showing statistical significance in ten out of 11 models. NU was a statistically significant predictor of survival under desiccation conditions in six out of 11 models, while mitochondrial haplotype was significant in only three. Among the first-order interactions, the genotype combination (MT:NU) stood out, being statistically significant in three models, while the interaction between nuclear background and sex (NU:sex) was significant in five models. Combinations of temperature with sex and nuclear background (NU:T and sex:T) were significant in two models. Other first-order interactions were significant in only one model each (MT:sex and MT:T). Among second-order interactions, it is important to note that the interactions of genotype with sex and temperature (MT:NU:T and MT:NU:sex) were significant in three quartet models each.

**Figure 3:**
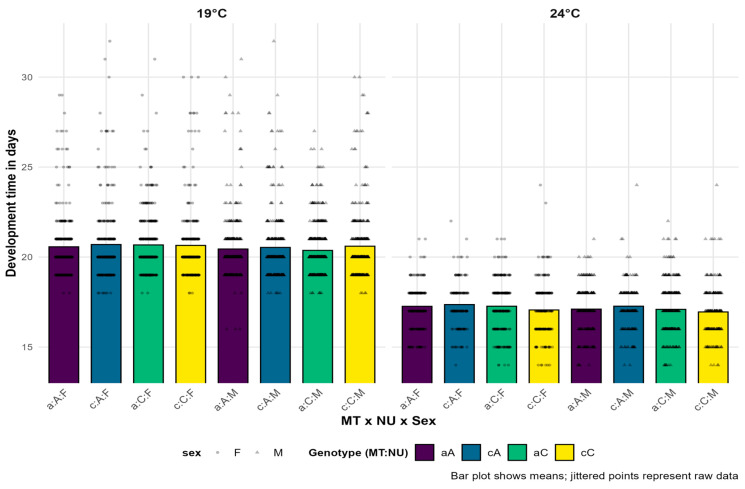
The mean developmental times of *D. subobscura* in days for all genotype and sex combinations across all 11 quartets combined at two experimental temperatures. Individual data points are overlaid as jittered dots, with shapes distinguishing between sexes. The lowercase letters represent the mtDNA haplotype (MT), where “a” corresponds to haplotype I, while “c” corresponds to haplotype II. The uppercase letters represent the corresponding nuclear background (NU), while F and M denote females and males (sex), respectively.

The mean development times of genotype combinations at the two experimental temperatures for the overall model are presented in Figure 3. Males and females exhibited similar development times across all quartets at a particular experimental temperature, with males having a slightly longer development overall. Individuals with different mito-nuclear haplotype combinations also had similar development times when considering all 11 quartets. No advantages of specific combinations for either sex or at either experimental temperature were observed. The most noticeable differences were between the two experimental temperatures, where it is clear that the experimental group at 24 °C developed on average 18% faster than the group at 19 °C. The results of the ANOVA for the GLM models of development time for the ten experimental quartets are given in Table 3, while the results of the ANOVA for the composite model are presented in Table 4.

The results show a small number of significant findings across the analyzed ten quartets, especially regarding interactions. As expected, temperature emerged as the most important factor in this experiment, as it was statistically significant in all ten quartet models as well as in the overall model. NU was the second most influential factor in the quartet models, showing statistical significance in five out of ten models. In the composite model, the only factor significant in addition to T was sex. This is somewhat surprising, as this factor was statistically significant in only two out of the ten quartet models. Mitochondrial haplotype was not significant in any model. Among interactions, it is worth noting that the genotype combination (MT:NU) was a good predictor of development time in three models where it was statistically significant. Females had faster development in some mito-nuclear genotype combinations, while in others, males developed faster. However, this effect was dependent on the quartet, so in the overall model, the interaction MT:NU:sex was not significant. Among other first-order interactions, only NU:T and NU:sex recorded a single statistically significant *p*-value in the models. Among higher-order interactions, only NU:sex:T had a significant *p*-value.

**Figure 4:**
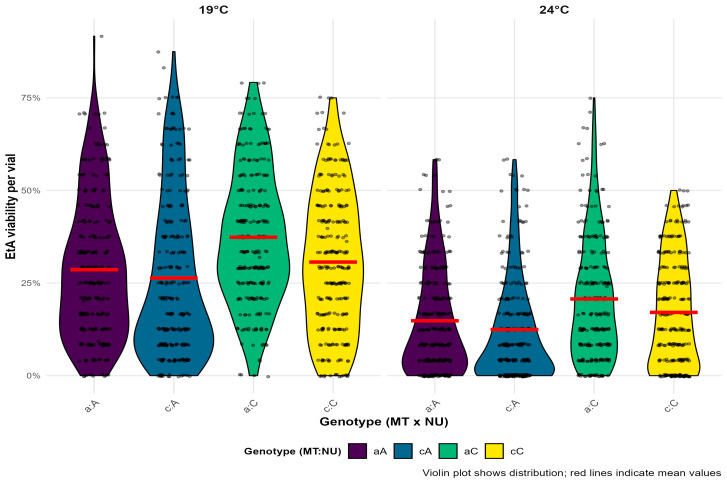
Violin plots showing the distribution of viability per vial EtA survival) for all genotype combinations across all 11 quartets combined on two experimental temperatures. Individual data points are overlaid as jittered dots. The mean viability for each group is indicated with a red horizontal line. Colors indicate genotype combinations. The lowercase letter represents the mtDNA haplotype (MT), where “a” corresponds to haplotype I, while “c” corresponds to haplotype II. The uppercase letters represent the corresponding nuclear background (NU).

The mean EtA viability values for the entire dataset are presented in Figure 4. From the violin plot of egg to adult survival, it can first be observed that individuals in the experimental group at 19 °C had an average of 14.5% higher survival compared with the experimental group at 24 °C. Generally, nuclear background C had 5.9% better survival than nuclear background A. At 24 °C, this difference was 5.26% in favor of C, while at 19 °C, background C survived 6.53% better than A. Interestingly, when comparing mitochondrial haplotypes, MTawas 4.5% more viable at 19 °C and 3% more viable at 24 °C. Thus, the mito-nuclear combination aC was the most successful in this experiment, followed by cC, then aA, while the combination cA showed the lowest viability at both temperatures. The results of the ANOVA for the GLM models of EtA survival for all 11 modeled quartets are presented in Table 5, while the results of the ANOVA for the overall model are given in Table 6.

T was a significant factor in all 11 quartet models as well as in the overall model. NU was statistically significant in nine out of 11 quartets and in the overall model. Interestingly, NU was the most influential factor for survival in five out of these nine quartets. MT significantly influenced viability in five out of 11 quartet models and was also significant in the overall model. Among first-order interactions, the MT:NU combination was the most significant, being significant in seven quartet models as well as in the overall model. The combination of MT and T was significant in two quartets, while the combination of NU and T significantly influenced survival in three out of 11 models, although none of these interactions were significant in the overall model. The interaction of all three factors (MT:NU:T) was statistically significant in three quartets but not in the overall model.

**Figure 5:**
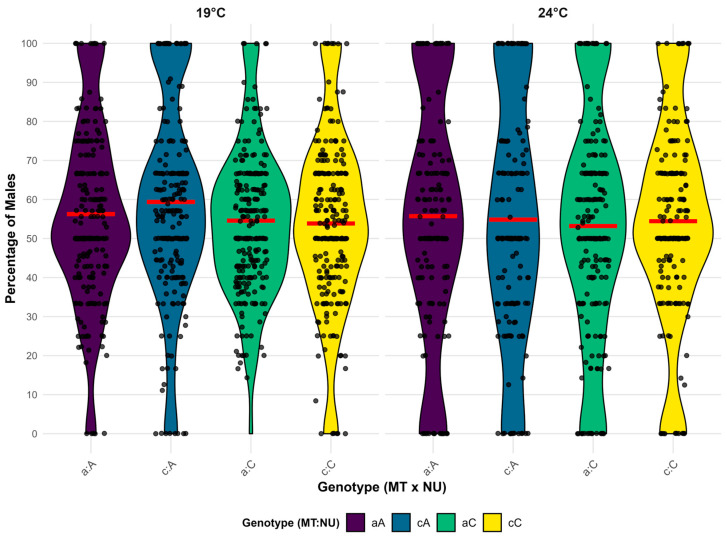
Violin plots showing the distribution of the percentage of males per vial for all genotype combinations across the whole dataset on two experimental temperatures. Individual data points are overlaid as jittered dots. The mean percentage of males for each group is indicated with a red horizontal line. Colors indicate genotype combinations. The lowercase letter represents the mtDNA haplotype (MT), where “a” corresponds to haplotype I, while “c” corresponds to haplotype II. The uppercase letters represent the corresponding nuclear background (NU).

The mean percentages of males per vial among hatched *D. subobscura* individuals in the experiments for the entire dataset are shown in Figure 5. All mito-nuclear combinations had a slightly higher percentage of males than the expected 50%. When considering the entire dataset, the average percentage of males per vial was 55.3%, which was statistically significantly different from the expected 50% (*p* < 0.001). A slightly higher percentage of males was recorded for NU A compared with nuclear background C, with about 2.6% more males on average per vial.

NU was the most influential factor on the sex ratio, with statistical significance in five quartets, while mitochondrial haplotype significantly influenced the sex ratio in only two models. T was not a significant factor in any quartet. Among factor combinations, only the interaction MT:NU was significant in one quartet. In the overall model, only temperature was significant. The ANOVA results for the fitness component percentage of males for the eleven quartets are given in Table 7, while the results of the ANOVA for the overall model are presented in Table 8. Since deviations from the expected 50% males were not large, this component did not show a large number of statistically significant factors or their interactions.

## 4. Discussion

The first analyzed life-history component was resistance to desiccation, which represents one of the most significant environmental stressors species encounter in their natural habitat. Although desiccation resistance and resistance to other stresses cannot be considered life-history traits in the strict sense, they still significantly contribute to the survival of individuals [57]. Today, with global warming being a research focus more than ever, the resistance of individuals to withstand extremely arid conditions is a crucial component of adaptive value [58]. The development time is a particularly important component of fitness in species with overlapping generations [59]. In the *Drosophila* genus, faster development is considered an adaptive advantage, as these species in nature lay eggs in fermenting fruit, which is an ephemeral habitat of limited duration [60,61]. Additionally, since the fruit in which the individuals develop is in the process of decay and may also be consumed by herbivores, individuals with faster development are thought to have an advantage.

Temperature (along with the light regime) emerged as the most influential factor in all analyzed life history traits. The significant role of temperature in the fitness component of desiccation resistance was expected, as it is known that metabolism is faster at higher temperatures, and all living organisms lose water more rapidly as temperature increases [62]. Insects, as poikilothermic organisms, depend on external temperature to drive enzymes involved in the chemical reactions necessary for their complex development [63]. Therefore, the fact that temperature is a crucial factor in development time should not be surprising. Additionally, it is known that insects cannot complete their development below or above certain temperature thresholds, defining the upper and lower limits, as well as the optimal temperature for the development and survival of insect species [64]. Thus, the role and significance of temperature in the experiments on survival from the egg to the adult stage is clear. Sex also proved to be an important factor influencing survival under desiccation stress in our model species. Across all models, females consistently exhibited greater resilience to arid conditions, which is consistent with established sexual dimorphism in this trait. Females’ ability to withstand desiccation conditions better is largely attributed to morphological traits, primarily larger body size [65,66,67,68,69]. A larger body implies a smaller surface-to-volume ratio [70], making them less exposed to environmental conditions and able to retain water better under desiccation conditions [69]. On the other hand, in the development time experiment, sex was significant but not to the same extent as in the desiccation experiment. Though development time showed moderate sexual differentiation (1% female advantage, *p* = 0.0037), this aligns with documented 2–4% dimorphism in congeneric species [71,72,73,74]. In contrast, in *D. obscura* [75], males were observed to develop 0.12% faster, but this difference was not statistically significant. In all experiments, the nuclear background had a greater impact than the mitochondrial haplotype, which is expected given the incomparably greater complexity and amount of DNA information contained in the nuclear genome compared with the mitochondrial genome.

### 4.1. Influence of Analyzed Factors on Life History Traits in D. subobscura

The theoretical assumption from the work of Christie [43] that the reason for the annual fluctuations in the frequencies of the two most common mitochondrial haplotypes is their adaptation to different temperatures was not confirmed in the experiments conducted within this paper. When considering the entire dataset, the two haplotypes, as well as their specific mito-nuclear combinations, did not show adaptation to a specific temperature. Fitness outcomes varied across quartets, with some showing one haplotype outperforming at higher temperatures and others showing the opposite, making it impossible to draw general conclusions. In the literature, studies have found significant differences in the adaptive advantage of one of the two mitochondrial haplotypes in the context of specific life history components. For example, Castro [40] showed higher fertility in individuals with haplotype I. Conversely, Christie [41], using the same experimental lines, demonstrated the superiority of haplotype II in desiccation resistance, survival from larva to adult, and development time. It is important to note that these experiments did not test mito-nuclear combinations but only mtDNA haplotypes on their original nuclear backgrounds. However, the same group of authors was unable to confirm the results from these two studies, as they did not observe adaptive advantages of haplotypes I and II on a uniform nuclear background in different life-history traits [42]. The only analyzed component in which one of the haplotypes showed a statistically significant advantage in this paper was egg to adult viability, where individuals with haplotype I (a) showed a slightly higher survival rate. Interestingly, in terms of survival, individuals with nuclear background C were far more successful than those with nuclear background A, contributing to aC being the most viable genotypic combination.

The viability experiment was the only one where these two factors were significant in the overall model, as well as in approximately half of the quartet models (5/11 and 7/11). While the overall models for other components showed no significance of mito-nuclear interactions and mtDNA variability on fitness, their effects were notable in several individual quartets. Although this variability likely does not imply fundamental differences between the two main haplotypes, it does suggest differences specific to the combinations of haplotypes within particular quartets, i.e., variability across the entire mtDNA. This aligns with broader research refuting the strict neutrality of mtDNA [5,6,7,8,10,11], particularly showing the adaptive significance of sympatric mtDNA sequence variability [20,27,75].

In general, across almost all analyzed life history components, the MT:NU interaction was significant in more models than mtDNA as a factor, showing that mito-nuclear interactions are more important as units of selection than mtDNA haplotypes themselves, consistent with previously published works [23,24,74,75]. The two investigated mtDNA haplotypes differ by one substitution in the *ND5* gene and one mutation in the gene encoding the small subunit of mitochondrial ribosomal RNA (12S RNA) [20]. A mutation within the 12S rRNA gene appears to be the primary site of action for a form of balancing selection, as this RNA molecule, along with 16S RNA and a large number of nuclear-encoded proteins, forms the mitoribosome. These riboprotein structures are responsible for the translation of mtDNA-encoded proteins, which underpin mitochondrial metabolic processes that must influence individual fitness. Since this crucial cellular process depends on the close interaction of the two genomes [76], it is not surprising that MT:NU interactions represent the basic level of selection. Mutations in ribosomal DNA can significantly compromise fitness if their RNA product is incompatible with nuclear proteins, leading to selection against them. The existence of polymorphisms in these genes implies the presence of corresponding nuclear variants compatible with mtDNA polymorphisms, indicating the existence of mito-nuclear coadaptations maintained by selection mechanisms. Between individual lines, there are likely additional differences in sequences in parts of the genome that were not genotyped, so other levels of interaction may also be significant. The significance of the MT:NU interaction within individual quartet models indicates the presence of mito-nuclear interactions at these specific sites.

Although a large number of experimental studies confirm the significant effect of mito-nuclear interactions on individual fitness [23,24,77,78,79,80,81,82,83,84,85], most have analyzed interpopulation variability [23,78,86,87,88]. Some have even examined trans-species mito-nuclear hybrids [74,89,90,91,92] to observe mito-nuclear effects better using greater divergence between lines. Few studies have examined the effects of mitochondrial variability within populations [23,26,27,93], and their results were not consistent across experimental blocks, observing the magnitude of these effects comparable to the results published here. Very similar research on the adaptive significance of sympatric mtDNA variation in a closely related species, *D. obscura*, was published recently [75], with the experiments conducted analyzing the same life history components as here. However, there is a notable difference in the mtDNA variation in the natural populations of the two related species. Unlike *D. subobscura*, where the two most common mtDNA haplotypes are evenly distributed across the species’ range [30,33], in *D. obscura*, there is a geographic structuring with the frequencies of the two main haplogroups drastically different between Eastern and Western Europe [75]. The variability is significantly lower in the eastern part of the range. The experiments on *D. subobscura* revealed fewer clear trends in life history traits, as *D. obscura* models showed a greater significance of both mtDNA haplotypes and mito-nuclear interactions on fitness components, with both factors being significant in more than half of models for all components except the percentage of males. Interestingly in both species, these two factors had the biggest influence on viability. Additionally, both studies agree that mito-nuclear interactions are the primary units of selection, as a greater number of models in both species demonstrated the significance of mito-nuclear interactions compared with the mitochondrial haplotype alone.

When it comes to comparing sympatric haplotypes, especially mitochondrial haplotypes I and II, and their mito-nuclear interactions on the model species used here, it is important to mention the results of two studies [26,27]. First [27], all four components examined in this work (desiccation resistance, development time, viability from egg to adult, and the percentage of males), as well as longevity, were analyzed, while the second [26] measured the metabolic rate of the same lines. It is worth highlighting the difference in experimental design between these two works and our paper, as they [26,27] did not have a complete comparison design. In the first experimental block, they compared haplotype I with the rare haplotype D; in the second block, haplotype II with the rare haplotype D, while in the third block, they compared haplotypes I and II exclusively on the nuclear background D. The first two blocks had a complete design, i.e., all four mito-nuclear combinations (e.g., I^I^, I^D^, D^I^, and D^D^ in the first block), while the third block was incomplete, comparing only DI and DII, so this block had only the effect of the mtDNA haplotype, not the MT:NU interaction. Both discussed studies found a weak effect of mtDNA and mito-nuclear variability on fitness, having these two factors significant in only a few blocks across all experiments. Moreover, these effects were not based on differences between the two main haplotypes, but between the main and the rare haplotype D. This finding fully corresponds to the results in this paper in all components except viability, where we found a greater influence of mtDNA and mito-nuclear interactions. The effects of mtDNA and MT:NU were recorded only at specific lines, indicating the adaptive significance of variability present in those specific lines rather than differences separating haplotypes I and II.

Overall, across all analyzed life-history components in this paper, mito-nuclear hybrids did not show lower fitness compared with mtDNA haplotypes on their own background, as would be expected and in line with numerous literature datasets [82,87,94,95,96]. A possible reason for this is that in most of the cited studies, mito-nuclear lines were formed by crossing lines from very distant populations, unlike our experimental lines, where all crossed lines originated from the same population. In certain quartet comparisons for a specific life history component, mito-nuclear hybrids had the lowest recorded fitness, while in other quartets, the lowest fitness was observed in mtDNA haplotypes on their own nuclear backgrounds, so no trend could generally be observed in the composite models.

### 4.2. Sex-Specific Effects on Mitochondrial and Mito-Nuclear Variability

In addition to exploring the adaptive significance of intrapopulation mtDNA variability and the influence of mito-nuclear interactions on fitness, this study aimed to assess the role of two forms of balancing selection in maintaining mtDNA diversity. Specifically, the impact of sex-specific selection was investigated through interactions between sex and mtDNA haplotype (MT:sex) and sex and mito-nuclear combinations (MT:NU:sex) in desiccation resistance and development time. For the fitness component of the percentage of males, sex-specific effects were measured indirectly, as the proportion of one sex was evaluated under the assumption of a 1:1 sex ratio, inherently linking mtDNA and mito-nuclear effects to sex-specific fitness differences.

The signature of sex-specific selection was observed only in a few quartets, likely indicating that this type of balancing selection does not maintain high frequencies of the two main haplotypes but rather acts on sites specific to each quartet. These findings align with previous studies on *D. subobscura* using sympatric haplotypes [26,27], where a weak signature of this type of balancing selection was also observed, indicating interactions were significant in a few sporadic models that compared either of the haplotypes I and II with a rare haplotype D across all tested life history traits. The obtained result is also somewhat consistent with previous empirical results in other model species, such as *D. melanogaster* and *Acanthoscelides obtectus*, depending on the type of lines used in the study (sympatric or allopatric), resulting in more or less significant effects [23,97,98,99,100]. When comparing the results of this study to previous work on *D. obscura* [75], the findings for development time and the percentage of males are consistent, both revealing a weak signature of sex-specific selection, though with slightly fewer significant *p*-values in the *D. subobscura* models. However, for desiccation resistance, a strong contrast is observed: *D. obscura* exhibits a very strong signal of sex-specific selection, whereas, in *D. subobscura*, only a few quartet models had these significant interactions.

### 4.3. Interaction of Variable Environment and Genotype on Adaptive Value

In addition to sex-specific selection, this paper also examines the adaptive value of different genotypes under varying environmental conditions. To discern the role of environment-dependent selection in maintaining intrapopulation mtDNA variability, interactions of MT:T and MT:NU:T were analyzed when modeling all fitness components. The different experimental temperature regimes also implied varying lengths of light and dark periods. Our results show that the differential fitness of mtDNA haplotype bearers depending on experimental temperature (MT:T) was significant only in a few quartet models. The temperature-specific effect of mito-nuclear interactions was observed only in the desiccation resistance experiment, where the overall model had a significant MT:NU:T interaction, similar to several quartet models in the desiccation resistance and survival experiments.

The interactions between temperature and mito-nuclear genotype have been examined in a wide range of organisms, although most studies have focused on mito-nuclear lines derived from different populations. A group of scientists led by Burton [77,79] used interpopulation hybrids of *Tigriopus californicus* as a model organism to demonstrate that variations in environmental factors, such as temperature and light, can be responsible for maintaining mito-nuclear variability. It is significant to mention the three studies [24,80,85] that used the same experimental lines of the model species *Callosobruchus maculatus* and, through various experiments on life history components and competition, confirmed the temperature specificity of mito-nuclear interactions. These three studies, similar to most others in this field, conducted experiments on lines derived from different populations. Although the specific experimental lines used originated from five different populations, it was determined that all mtDNA haplotypes used in the studies naturally occur sympatrically in West African populations of this species [101]. Therefore, although they used a completely different model system compared with this paper, the magnitude of effects in these studies is similar to this paper, especially in light of the natural segregation of these haplotypes and the similarity of experimental setups and analyzed components.

When it comes to studies on *Drosophila* species, the only comparable work regarding environment-by-genotype interactions using sympatric mtDNA variation was conducted on *D. obscura* [75]. Interestingly, in *D. obscura,* genotype-by-environment interactions had a much bigger influence on the adaptive values than in the results presented in this paper for all four life history components assayed. In both model species percentage of males and developmental time showed the weakest signatures of this type of balancing selection, and while *D. subobscura* showed no significant genotype-by-environment interactions at all, the experiments on *D. obscura* [75] had a few models with those interactions significant. Moreover, while the viability and desiccation resistance showed weak to medium signals of genotype-by-environment interactions in *D. subobscura*, the previously published work on *D. obscura* [75] showed very strong influences of the two interactions that measure this type of balancing selection in these two life-history components. The reason behind the bigger impact of environment-by-genotype interactions on fitness in *D. obscura* may be the variability that is present in the natural populations of this species. The two main mitochondrial haplogroups, although present throughout Europe, have very different frequencies in the eastern and western parts of its range [102], which may be due to the adaptations to different environmental conditions. Also, it is worth noting that in *D. subobscura,* variability in experimental lines was minimized by choosing only lines with RSA haplotypes I and II and main *Cyt b* haplotype, while in *D. obscura,* a wide range of haplotypes was chosen, with some experimental blocks consisting of most distant haplotypes that were recorded in natural populations [75]. In addition to temperature as the most significant external factor, studies on *Drosophila* species have also used various experimental setups with variability in diet and oxygen levels [84,103], showing significant effects of these environmental factors on mito-nuclear variability.

The results indicate a greater importance of environment-dependent selection compared with sex-specific selection (SSS) in maintaining sympatric mitochondrial variability in this specific model species. The absence of SSS signals between the two main mitochondrial haplotypes suggests that environment-dependent selection could play a minor, secondary role to negative frequency-dependent selection, which is probably the main selective force involved in maintaining the frequencies of the two mtDNA haplotypes of *D. subobscura* [20,104], likely mediated through mito-nuclear interactions. Kurbalija Novičić [26] proposed negative frequency-dependent selection (NFDS) as the mechanism responsible for maintaining the equilibrium frequencies of the two mitochondrial haplotypes of *D. subobscura* in nature. First, theoretical simulations showed that the observed dynamics of haplotype frequencies I and II in nature are consistent with the NFDS mechanism [104], and this hypothesis was experimentally confirmed a few years later [20].

## 5. Conclusions

In general, the experiments conducted within this paper demonstrate the complexity of maintaining intrapopulation mtDNA variability. Results across all life-history components show that sex-specific selection is not responsible for maintaining stable frequencies of mitochondrial haplotypes I and II in natural populations of *Drosophila subobscura*. The desiccation resistance experiment shows that environment-by-genotype interactions could have a role in maintaining two mtDNA haplotypes in equilibrium, acting on the joint mito-nuclear genotype. Moreover, the effects of sex-specific and environment-specific selection were observed when comparing haplotypes within independent quartets. Thus, the results of this study highlight the importance of these balancing selection mechanisms in the broader context of maintaining sympatric mtDNA variability. The role of each type of balancing selection likely depends on the specific nucleotide substitutions that differentiate the haplotypes within the quartets.

## Figures and Tables

**Table 1:** The influence of mitochondrial haplotype (MT), nuclear background (NU), sex, temperature (T), and their interactions on desiccation resistance. The results of the overall model, which included the entire dataset, are presented. df—degrees of freedom; F.ratio—*F* statistic; Chisq—Chi-square value; strata—the model is stratified by that variable; *p*-values significant at *p* < 0.05 are shown in bold.

	Overall Model				
	df1	df2	F.Ratio	Chisq	*p*
**MT**	1	Inf	0.123	0.123	0.7261
**NU**	1	Inf	15	15	**0.0001**
**sex**	1	Inf	228	228	**<0.0001**
**T**			strata		
**MT:NU**	1	Inf	0.011	0.011	0.9148
**MT:sex**	1	Inf	0.444	0.444	0.5051
**MT:T**	1	Inf	1	1	0.2261
**NU:sex**	1	Inf	5	5	**0.0214**
**NU:T**	1	Inf	18	18	**<0.0001**
**sex:T**	1	Inf	7	7	**0.0086**
**MT:NU:sex**	1	Inf	0.011	0.011	0.9149
**MT:NU:T**	1	Inf	7	7	**0.0075**
**MT:sex:T**	1	Inf	0.108	0.108	0.742
**NU:sex:T**	1	Inf	0.789	0.789	0.3744
**MT:NU:sex:T**	1	Inf	2	2	0.188

**Table 2:** The influence of mitochondrial haplotype (MT), nuclear background (NU), temperature (T), sex, and their interactions on desiccation resistance. The results of 11 quartet models (Q1–Q11) are presented. Loglik—Log-likelihood; Chisq—Chi-square value; df—degrees of freedom; strata—the model is stratified by that variable; *p*-values significant at *p* < 0.05 are shown in bold.

	**Q1**				**Q2**				**Q3**			
	**Loglik**	**Chisq**	**df**	***p***	**Loglik**	**Chisq**	**df**	***p***	**Loglik**	**Chisq**	**df**	***p***
**MT**	−1148.19	4.8803	1	**0.0272**	−1159.8	0.5528	1	0.4572	−1189.46	0.0343	1	0.853
**NU**	−1143.51	9.3656	1	**0.0022**	−1149.2	21.3178	1	**<0.0001**	−1181.05	16.8154	1	**<0.0001**
**sex**	−1141.16	4.6976	1	**0.0302**	−1116.2	65.9859	1	**<0.0001**	−1178.67	4.7588	1	**0.0291**
**T**	−1012.25	257.8278	1	**<0.0001**		strata			−1015.66	326.0198	1	**<0.0001**
**MT:NU**	−1010.51	3.4829	1	0.062	−1114.3	3.7613	1	0.0524	−1004.6	22.1343	1	**<0.0001**
**MT:sex**	−1009.8	1.413	1	0.2346	−1113.7	1.1981	1	0.2737	−1003.45	2.2894	1	0.1303
**NU:sex**	−1008.93	1.7341	1	0.1879	−1104.1	19.1948	1	**<0.0001**	−1002.11	2.6767	1	0.1018
**MT:T**	−1003.17	11.5261	1	**0.0007**	−1103.5	1.2358	1	0.2663	−1001.5	1.2239	1	0.2686
**NU:T**	−1002.11	2.122	1	0.1452	−1102.1	2.8702	1	0.0902	−999.75	3.5015	1	0.0613
**sex:T**	−1000.6	3.0165	1	0.0824	−1101	2.1064	1	0.1467	−999.75	0.0082	1	0.9278
**MT:NU:sex**	−1000.58	0.0429	1	0.8358	−1098.4	5.2707	1	**0.0217**	−999.66	0.1791	1	0.6722
**MT:NU:T**	−998.17	4.8132	1	**0.0282**	−1098.4	0.0042	1	0.9483	−997.46	4.3989	1	**0.036**
**MT:sex:T**	−997.7	0.935	1	0.3336	−1098.3	0.1394	1	0.7088	−997.09	0.7316	1	0.3924
**NU:sex:T**	−997.69	0.0202	1	0.8869	−1098.1	0.3369	1	0.5616	−996.34	1.4953	1	0.2214
**MT:NU:sex:T**	−996.96	1.4783	1	0.224	−1095.4	5.4394	1	**0.0197**	−995.59	1.5061	1	0.2197
	**Q4**				**Q5**				**Q6**			
	**Loglik**	**Chisq**	**df**	***p***	**Loglik**	**Chisq**	**df**	***p***	**Loglik**	**Chisq**	**df**	***p***
**MT**	−1313.2	10.3326	1	**0.0013**	−1250.9	0.0565	1	0.8121	−1138.19	2.7669	1	0.0962
**NU**	−1310.5	5.4985	1	**0.019**	−1250.1	1.5183	1	0.2179	−1137.86	0.656	1	0.418
**sex**	−1305.9	9.1364	1	**0.0025**	−1247.9	4.4387	1	**0.0351**	−1132.59	10.5357	1	**0.0012**
**T**	−1134.3	343.3287	1	**<0.0001**	−1088.5	318.7242	1	**<0.0001**	−998.94	267.3056	1	**<0.0001**
**MT:NU**	−1132.9	2.7134	1	0.0995	−1087.3	2.4265	1	0.1193	−996.17	5.5454	1	**0.0185**
**MT:sex**	−1132.3	1.2293	1	0.2675	−1084.1	6.3354	1	**0.0118**	−996.05	0.2371	1	0.6263
**NU:sex**	−1131.8	0.9489	1	0.33	−1073.2	21.8891	1	**<0.0001**	−995.81	0.4653	1	0.4951
**MT:T**	−1131.4	0.8734	1	0.35	−1071.9	2.5354	1	0.1113	−995.43	0.7653	1	0.3817
**NU:T**	−1131.2	0.3108	1	0.5772	−1070.2	3.5676	1	0.0589	−995.4	0.0663	1	0.7968
**sex:T**	−1131.1	0.1961	1	0.6579	−1067.7	4.9505	1	**0.0261**	−994.42	1.9549	1	0.1621
**MT:NU:sex**	−1131.1	0.0787	1	0.7791	−1065.6	4.169	1	**0.0412**	−994.13	0.5917	1	0.4418
**MT:NU:T**	−1130.7	0.7019	1	0.4022	−1064.4	2.4008	1	0.1213	−993.76	0.7364	1	0.3908
**MT:sex:T**	−1129.8	1.8217	1	0.1771	−1061.9	4.963	1	**0.0259**	−992.88	1.7559	1	0.1851
**NU:sex:T**	−1129.5	0.5949	1	0.4405	−1061	1.7083	1	0.1912	−991.8	2.1631	1	0.1414
**MT:NU:sex:T**	−1127.8	3.3377	1	0.0677	−1061	0.0487	1	0.8253	−991.41	0.7705	1	0.3801
	**Q7**				**Q8**				**Q9**			
	**Loglik**	**Chisq**	**df**	***p***	**Loglik**	**Chisq**	**df**	***p***	**Loglik**	**Chisq**	**df**	***p***
**MT**	−1200	0.2367	1	0.6266	−1095.8	19.6151	1	**<0.0001**	−1329.5	0.309	1	0.5783
**NU**	−1199.7	0.5925	1	0.4414	−1095.8	0.0247	1	0.8752	−1329.4	0.3315	1	0.5648
**sex**	−1164.6	70.1205	1	**<0.0001**	−1086.6	18.4691	1	**<0.0001**	−1329.4	0.0216	1	0.883
**T**		strata				strata			−1170.9	316.9546	1	**<0.0001**
**MT:NU**	−1164.6	0.0743	1	0.7852	−1086.6	0.0022	1	0.9627	−1170.5	0.7292	1	0.3931
**MT:sex**	−1164.5	0.0846	1	0.7711	−1086.5	0.2363	1	0.6269	−1170.3	0.3765	1	0.5395
**NU:sex**	−1161.2	6.7146	1	**0.0096**	−1084.3	4.2372	1	**0.0396**	−1166.8	6.9931	1	**0.0082**
**MT:T**	−1161.2	0.0038	1	0.9509	−1083.4	1.9403	1	0.1636	−1166.8	0.017	1	0.8961
**NU:T**	−1160.3	1.6376	1	0.2007	−1083.4	0.011	1	0.9165	−1165.2	3.3354	1	0.0678
**sex:T**	−1160.1	0.4188	1	0.5175	−1082.2	2.3557	1	0.1248	−1162.3	5.7705	1	**0.0163**
**MT:NU:sex**	−1160.1	0.0349	1	0.8518	−1082.2	0.014	1	0.9059	−1159.5	5.6216	1	**0.0177**
**MT:NU:T**	−1155	10.3328	1	**0.0013**	−1081.8	0.6822	1	0.4088	−1158.9	1.0722	1	0.3004
**MT:sex:T**	−1154.9	0.0795	1	0.778	−1081.7	0.3684	1	0.5438	−1155.5	6.9442	1	**0.0084**
**NU:sex:T**	−1153.2	3.3669	1	0.0665	−1081.6	0.0517	1	0.8201	−1155.3	0.2481	1	0.6184
**MT:NU:sex:T**	−1152.5	1.451	1	0.2284	−1081.6	0.0104	1	0.9187	−1155.3	0.0087	1	0.9257
	**Q10**				**Q11**							
	**Loglik**	**Chisq**	**df**	***p***	**Loglik**	**Chisq**	**df**	***p***				
**MT**	−1284.4	0.2943	1	0.5875	−1209.8	0.7311	1	0.3925				
**NU**	−1279.7	9.5585	1	**0.002**	−1200.5	18.5688	1	**<0.0001**				
**sex**	−1263.2	32.9892	1	**<0.0001**	−1178.2	44.498	1	**<0.0001**				
**T**	−1142.1	242.0688	1	**<0.0001**		strata						
**MT:NU**	−1134.8	14.6483	1	**0.0001**	−1177.2	2.0752	1	0.1497				
**MT:sex**	−1134.7	0.1403	1	0.7079	−1177	0.3678	1	0.5442				
**NU:sex**	−1134.3	0.9127	1	0.3394	−1176.6	0.8225	1	0.3644				
**MT:T**	−1134	0.6498	1	0.4202	−1176.2	0.9272	1	0.3356				
**NU:T**	−1128.9	10.1564	1	**0.0014**	−1163.4	25.5359	1	**<0.0001**				
**sex:T**	−1127.2	3.355	1	0.067	−1162.5	1.7882	1	0.1811				
**MT:NU:sex**	−1126.3	1.7725	1	0.1831	−1161.4	2.2491	1	0.1337				
**MT:NU:T**	−1125.8	1.0604	1	0.3031	−1161.4	0.0024	1	0.9606				
**MT:sex:T**	−1125.7	0.1736	1	0.6769	−1161.3	0.1994	1	0.6552				
**NU:sex:T**	−1125.1	1.1011	1	0.294	−1158.9	4.6568	1	**0.0309**				
**MT:NU:sex:T**	−1124.8	0.783	1	0.3762	−1158.6	0.6739	1	0.4117				

**Table 3:** The influence of mitochondrial haplotype (MT), nuclear background (NU), sex, temperature (T). and their interactions on development time from egg to adult. The results of ten quartet models (Q1–Q6, Q8–Q11) are presented, as the seventh quartet was incomplete for statistical analysis. SSq—Sum of squares; F—*F* statistic; *p*-values significant at *p* < 0.05 are shown in bold.

	**Q1**			**Q2**			**Q3**		
	**SSq**	***F***	***p***	**SSq**	***F***	***p***	**SSq**	***F***	***p***
**MT**	1.235	1.1037	0.2979	6.622	2.9884	0.0924	0.028	0.0241	0.8772
**NU**	9.265	8.2821	**0.0056**	0.005	0.0024	0.961	17.663	15.0661	**0.0003**
**sex**	0.043	0.038	0.8455	1.752	0.7906	0.3747	2.966	2.5301	0.1125
**T**	208.098	186.0237	**<0.0001**	310.692	140.2148	**<0.0001**	213.226	181.879	**<0.0001**
**MT:NU**	12.813	11.4539	**0.0013**	17.389	7.8477	**0.0081**	0.711	0.6069	0.4396
**MT:sex**	1.944	1.7376	0.1883	7.598	3.4291	0.0651	1.714	1.4623	0.2273
**NU:sex**	1.042	0.931	0.3352	0.103	0.0465	0.8294	2.684	2.2897	0.131
**MT:T**	0.172	0.1541	0.6961	1.897	0.8563	0.3609	0.011	0.0096	0.9224
**NU:T**	1.231	1.1005	0.2986	5.381	2.4282	0.1279	0.273	0.2329	0.6314
**sex:T**	3.621	3.2373	0.0728	4.372	1.9733	0.1612	0.053	0.0456	0.831
**MT:NU:sex**	1.613	1.4418	0.2306	2.026	0.9145	0.3397	0.588	0.5015	0.4792
**MT:NU:T**	0.895	0.8005	0.3747	0.168	0.076	0.7844	0.089	0.076	0.784
**MT:sex:T**	2.523	2.255	0.1341	8.309	3.7499	0.0538	2.084	1.7774	0.1832
**NU:sex:T**	0	0.0002	0.9884	0.016	0.0071	0.9327	0.954	0.8134	0.3676
**MT:NU:sex:T**	0	0.0002	0.9892	0.008	0.0034	0.9534	0.039	0.0329	0.8561
	**Q4**			**Q5**			**Q6**		
	**SSq**	***F***	***p***	**SSq**	***F***	***p***	**SSq**	***F***	***p***
**MT**	0.11	0.0477	0.828	0.867	0.6604	0.4199	3.14	2.8573	0.096
**NU**	3.38	1.4831	0.2293	1.696	1.2913	0.2606	6.681	6.079	**0.0165**
**sex**	0.13	0.0559	0.8132	1.494	1.1376	0.2866	7.915	7.2024	**0.0077**
**T**	527.24	231.0767	**<0.0001**	156.829	119.4216	**<0.0001**	277.359	252.3781	**<0.0001**
**MT:NU**	2.75	1.2037	0.2781	0	0.0002	0.989	4.848	4.4112	**0.0398**
**MT:sex**	1.69	0.7391	0.3904	0.053	0.0403	0.841	0.051	0.0466	0.8293
**NU:sex**	0.08	0.0341	0.8536	0	0.0002	0.9894	3.393	3.087	0.0799
**MT:T**	1	0.4367	0.5119	0.311	0.2368	0.6284	2.033	1.8499	0.1788
**NU:T**	0.21	0.0906	0.7648	1.212	0.9229	0.3409	0.871	0.7924	0.3769
**sex:T**	0.03	0.0119	0.913	3.512	2.674	0.1026	2.689	2.4472	0.1187
**MT:NU:sex**	2.15	0.9408	0.3326	0.551	0.4196	0.5174	0.006	0.0057	0.94
**MT:NU:T**	0.44	0.1941	0.6615	0.016	0.0125	0.9112	1.287	1.1707	0.2835
**MT:sex:T**	0.21	0.0924	0.7613	1.136	0.8652	0.3527	0.053	0.0484	0.826
**NU:sex:T**	0.56	0.2437	0.6218	0.099	0.0752	0.784	2.01	1.8285	0.1773
**MT:NU:sex:T**	5.17	2.2652	0.133	1.102	0.8394	0.36	0	0.0004	0.9842
	**Q8**			**Q9**			**Q10**		
	**SSq**	***F***	***p***	**SSq**	***F***	***p***	**SSq**	***F***	***p***
**MT**	3.95	2.2987	0.1349	0.071	0.0477	0.8278	0.62	0.3205	0.5739
**NU**	0.17	0.0967	0.757	2.923	1.9643	0.1654	18.57	9.55	**0.0033**
**sex**	3.07	1.7864	0.1819	5.157	3.466	0.0642	0.55	0.2841	0.5943
**T**	569.86	331.2609	**<0.0001**	195.683	131.5183	**<0.0001**	336.33	172.9925	**<0.0001**
**MT:NU**	1.99	1.1572	0.2865	0.004	0.0025	0.9606	1.48	0.7621	0.387
**MT:sex**	0.82	0.4778	0.4897	0.141	0.0951	0.7582	4.39	2.2576	0.1335
**NU:sex**	0.3	0.1746	0.6762	0.857	0.5759	0.4489	6.49	3.3361	0.0683
**MT:T**	0.03	0.0189	0.8912	0.958	0.6439	0.425	1.44	0.7383	0.3945
**NU:T**	1.67	0.9693	0.3289	6.017	4.0438	**0.0481**	0.64	0.3267	0.5703
**sex:T**	0	0	0.9975	0.051	0.0345	0.8529	0.02	0.01	0.9206
**MT:NU:sex**	1.28	0.7443	0.3887	0.199	0.1339	0.7148	3.44	1.7709	0.1838
**MT:NU:T**	1.16	0.6739	0.415	0.726	0.4882	0.487	1.04	0.5342	0.4684
**MT:sex:T**	0.41	0.2412	0.6235	0.044	0.0293	0.8642	0.09	0.0441	0.8337
**NU:sex:T**	2	1.1599	0.282	0.505	0.3396	0.5607	10.43	5.3643	**0.0209**
**MT:NU:sex:T**	0.59	0.3402	0.5599	0.089	0.0598	0.807	0	0	0.9969
	**Q11**								
	**SSq**	***F***	***p***						
**MT**	0.006	0.0036	0.9524						
**NU**	7.098	4.1628	**0.0472**						
**sex**	7.042	4.1302	**0.0429**						
**T**	180.339	105.7654	**<0.0001**						
**MT:NU**	0.007	0.0043	0.9482						
**MT:sex**	3.223	1.8901	0.1701						
**NU:sex**	5.99	3.5128	**0.0393**						
**MT:T**	0.006	0.0033	0.9542						
**NU:T**	5.367	3.1477	0.0826						
**sex:T**	3.746	2.1972	0.1391						
**MT:NU:sex**	3.06	1.7947	0.1812						
**MT:NU:T**	0.051	0.0298	0.8636						
**MT:sex:T**	1.68	0.9852	0.3216						
**NU:sex:T**	5.397	3.1654	0.0761						
**MT:NU:sex:T**	1.439	0.8439	0.3589						

**Table 4:** The influence of mitochondrial haplotype (MT), nuclear background (NU), temperature (T), sex, and their interactions on the development time from egg to adult. The results of the overall model, which included the entire dataset, are presented. SSq—Sum of squares; F—*F* statistic; *p*-values significant at *p* < 0.05 are shown in bold.

Overall Model			
	SSq	*F*	*p*
**MT**	3.32	1.8655	0.1725
**NU**	1.63	0.9146	0.3393
**sex**	24.91	13.9832	**0.0002**
**T**	2945.97	1653.667	**<0.0001**
**MT:NU**	0.49	0.2726	0.6017
**MT:sex**	0.01	0.006	0.938
**NU:sex**	0	0.001	0.9745
**MT:T**	0.34	0.1897	0.6633
**NU:T**	0.32	0.1812	0.6705
**sex:T**	1.43	0.8015	0.3707
**MT:NU:sex**	2.06	1.1538	0.2828
**MT:NU:T**	0.3	0.1702	0.6801
**MT:sex:T**	0.33	0.1875	0.6651
**NU:sex:T**	0.03	0.0191	0.8901
**MT:NU:sex:T**	1.83	1.0245	0.3115

**Table 5:** The influence of mitochondrial haplotype (MT), nuclear background (NU), temperature (T), and their interactions on the survival rate from egg to adult. The results of eleven quartet models are presented. df—degrees of freedom; Dev.—deviance; *p*-values significant at *p* < 0.05 are shown in bold.

	**Q1**			**Q2**			**Q3**		
	**df**	**Dev.**	***p***	**df**	**Dev.**	***p***	**df**	**Dev.**	***p***
**MT**	1	0.202	0.6529	1	4.519	**0.0335**	1	3.16	0.0755
**NU**	1	13.86	**0.0002**	1	3.851	**0.0497**	1	3.888	**0.0486**
**T**	1	63.455	**<0.0001**	1	37.18	**<0.0001**	1	154.174	**<0.0001**
**MT:NU**	1	15.154	**<0.0001**	1	0.106	0.7445	1	51.49	**<0.0001**
**MT:T**	1	0.227	0.6338	1	2.602	0.1067	1	0	0.993
**NU:T**	1	0.084	0.7716	1	1.093	0.2959	1	1.139	0.2859
**MT:NU:T**	1	13.939	**0.0002**	1	4.117	**0.0425**	1	1.813	0.1782
	**Q4**			**Q5**			**Q6**		
	**df**	**Dev.**	***p***	**df**	**Dev.**	***p***	**df**	**Dev.**	***p***
**MT**	1	1.588	0.2076	1	1.789	0.1811	1	10.067	**0.0015**
**NU**	1	0.009	0.9263	1	9.761	**0.0018**	1	259.242	**<0.0001**
**T**	1	128.388	**<0.0001**	1	150.216	**<0.0001**	1	27.126	**<0.0001**
**MT:NU**	1	2.286	0.1306	1	163.519	**<0.0001**	1	2.821	0.093
**MT:T**	1	0.021	0.8843	1	0.164	0.6859	1	1.531	0.216
**NU:T**	1	2.249	0.1337	1	0.296	0.5866	1	0.968	0.3251
**MT:NU:T**	1	0.158	0.6907	1	18.042	**<0.0001**	1	0.001	0.971
	**Q7**			**Q8**			**Q9**		
	**df**	**Dev.**	***p***	**df**	**Dev.**	***p***	**df**	**Dev.**	***p***
**MT**	1	26.745	**<0.0001**	1	2.047	0.1525	1	5.3278	**0.021**
**NU**	1	100.46	**<0.0001**	1	212.116	**<0.0001**	1	0.4925	0.4828
**T**	1	201.487	**<0.0001**	1	191.14	**<0.0001**	1	20.0071	**<0.0001**
**MT:NU**	1	29.364	**<0.0001**	1	10.208	**0.0014**	1	24.2489	**<0.0001**
**MT:T**	1	1.684	0.1944	1	9.599	**0.0019**	1	2.0824	0.149
**NU:T**	1	0.049	0.8254	1	11.639	**0.0006**	1	11.3598	**0.0008**
**MT:NU:T**	1	0.232	0.6301	1	0.474	0.4912	1	2.1439	0.1431
	**Q10**			**Q11**					
	**df**	**Dev.**	***p***	**df**	**Dev.**	***p***			
**MT**	1	56.06	**<0.0001**	1	1.59	0.2073			
**NU**	1	349.21	**<0.0001**	1	112.038	**<0.0001**			
**T**	1	80.64	**<0.0001**	1	57.671	**<0.0001**			
**MT:NU**	1	1.53	0.2155	1	9.782	**0.0018**			
**MT:T**	1	1.18	0.2783	1	4.777	**0.0288**			
**NU:T**	1	11.17	**0.0008**	1	1.742	0.1868			
**MT:NU:T**	1	0.68	0.4083	1	0.61	0.4348			

**Table 6:** The influence of mitochondrial haplotype (MT), nuclear background (NU), temperature (T), and their interactions on the EtA survival rate. The results of the overall model, which included the entire dataset, are presented. df—degrees of freedom; F.ratio—*F* statistic; Chisq—Chi-square value; *p*-values significant at *p* < 0.05 are shown in bold.

Overall Model					
	df1	df2	F.Ratio	Chisq	*p*
**MT**	1	Inf	31.78	31.78	**<0.0001**
**NU**	1	Inf	135.947	135.947	**<0.0001**
**T**	1	Inf	846.195	846.195	**<0.0001**
**MT:NU**	1	Inf	8.754	8.754	**0.0031**
**MT:T**	1	Inf	3.439	3.439	0.0637
**NU:T**	1	Inf	0.927	0.927	0.3355
**MT:NU:T**	1	Inf	2.515	2.515	0.1128

**Table 7:** The influence of mitochondrial haplotype (MT), nuclear background (NU), temperature (T), and their interactions on the percentage of males. The results of eleven quartet models (Q1–Q11) are presented. df—degrees of freedom; Dev.—deviance; Resid. df—residual degrees of freedom; Resid. Dev—residual deviance; *p*-values significant at *p* < 0.05 are shown in bold.

	**Q1**			**Q2**			**Q3**		
	**df**	**Dev.**	***p***	**df**	**Dev.**	***p***	**df**	**Dev.**	***p***
**MT**	1	3.9363	**0.0473**	1	4.2303	**0.0397**	1	0.0273	0.8687
**NU**	1	11.5675	**0.0007**	1	0.0542	0.8159	1	3.3019	0.0692
**T**	1	1.8604	0.1726	1	3.3982	0.0653	1	0.7101	0.3994
**MT:NU**	1	0.5187	0.4714	1	0.5907	0.4422	1	4.8891	**0.027**
**MT:T**	1	0.175	0.6757	1	1.3956	0.2375	1	0.1929	0.6605
**NU:T**	1	0.0508	0.8217	1	0.4416	0.5063	1	0.1309	0.7175
**MT:NU:T**	1	0.0045	0.9466	1	0.8013	0.3707	1	3.7121	0.054
	**Q4**			**Q5**			**Q6**		
	**df**	**Dev.**	***p***	**df**	**Dev.**	***p***	**df**	**Dev.**	***p***
**MT**	1	0.05857	0.8088	1	0.8918	0.345	1	0.342	0.5587
**NU**	1	0.36087	0.548	1	0.4435	0.5054	1	4.266	**0.0389**
**T**	1	0.11362	0.7361	1	0.5795	0.4465	1	0.1025	0.7489
**MT:NU**	1	0.2693	0.6038	1	0.9334	0.334	1	1.6035	0.2054
**MT:T**	1	0.22644	0.6342	1	3.5922	0.058	1	0.5781	0.4471
**NU:T**	1	0.95637	0.3281	1	0.3468	0.5559	1	1.1977	0.2738
**MT:NU:T**	1	0.12659	0.722	1	0.0168	0.8968	1	0.0237	0.8778
	**Q7**			**Q8**			**Q9**		
	**df**	**Dev.**	***p***	**df**	**Dev.**	***p***	**df**	**Dev.**	***p***
**MT**	1	3.7449	0.053	1	0.4742	0.4911	1	0.10195	0.7495
**NU**	1	8.4893	**0.0036**	1	8.9406	**0.0028**	1	0.01079	0.9173
**T**	1	1.817	0.1777	1	3.5683	0.0589	1	0.0054	0.9414
**MT:NU**	1	1.8395	0.175	1	1.051	0.3053	1	2.13222	0.1442
**MT:T**	1	0.2512	0.6162	1	0.3211	0.571	1	0.00283	0.9576
**NU:T**	1	0.2616	0.609	1	0.0426	0.8365	1	0.75705	0.3843
**MT:NU:T**	1	0.3482	0.5552	1	3.287	0.0698	1	1.03348	0.3093
	**Q10**			**Q11**					
	**df**	**Dev.**	***p***	**df**	**Dev.**	***p***			
**MT**	1	0.27254	0.6016	1	0.8806	0.348			
**NU**	1	0.27058	0.6029	1	5.1091	**0.0238**			
**T**	1	1.45227	0.2282	1	0.0172	0.8957			
**MT:NU**	1	1.01817	0.313	1	0.4506	0.5021			
**MT:T**	1	1.70655	0.1914	1	0.0369	0.8476			
**NU:T**	1	0.04034	0.8408	1	0.7592	0.3836			
**MT:NU:T**	1	2.63237	0.1047	1	0.3498	0.5542			

**Table 8:** The influence of mitochondrial haplotype (MT), nuclear background (NU), temperature (T), and their interactions on the percentage of males. The results of the overall model, which included the entire dataset, are presented. df—degrees of freedom; F.ratio—*F* statistic; Chisq—Chi-square value; *p*-values significant at *p* < 0.05 are shown in bold.

Overall Model					
	df1	df2	F.Ratio	Chisq	*p*
**MT**	1	Inf	0.447	0.447	0.5039
**NU**	1	Inf	1.956	1.956	0.162
**T**	1	Inf	5.124	5.124	**0.0236**
**MT:NU**	1	Inf	0.161	0.161	0.6879
**MT:T**	1	Inf	0.062	0.062	0.8034
**NU:T**	1	Inf	0.009	0.009	0.9261
**MT:NU:T**	1	Inf	0.601	0.601	0.4383

## Data Availability

Raw data are provided in spreadsheets, and can be downloaded at Supplementary Materials: Desication_resistance_raw.csv; development_time_raw.csv; viability_raw.csv.

## Supplementary Materials

The following supporting information can be downloaded at: https://www.mdpi.com/article/10.3390/insects16040415/s1, Figure S1: Egg-to-Pupa viability plot; Figure S2: Pupa-to-Adult viability plot; Table S1: Egg-to-Pupa quartet models; Table S2: Egg-to-Pupa overall model; Table S3: Pupa-to-Adult quartet models; Table S4: Pupa-to-Adult overall model.

## Abbreviations

The following abbreviations are used in this manuscript:

mtDNA	Mitochondrial DNA
MNIL	Mito-nuclear introgression line
NFDS	Negative frequency-dependent selection
SSS	Sex-specific selection
RSA	Restriction site analysis
*Cyt b*	Cytochrome b
IFL	Isofemale line
PCR	Polymerase chain reaction
RFLP	Restriction fragment length polymorphism
EtP	Egg-to-pupa
EtA	Egg-to-adult
PtA	Pupa-to-adult
MT	Mitochondrial haplotype
NU	Nuclear background
T	Temperature
ANOVA	Analysis of variance
REML	Reduced maximum likelihood
GLM	Generalized linear model
GLMM	Generalized linear mixed model
