# Supplementary figures and images for: Mechanisms Maintaining Mitochondrial DNA Polymorphisms: The Role of Mito-Nuclear Interactions, Sex-Specific Selection, and Genotype-by-Environment Interactions in Drosophila subobscura
Source: Insects. 2025 Apr 15;16(4):415. doi: 10.3390/insects16040415 (PMC12027999; doi:10.3390/insects16040415)

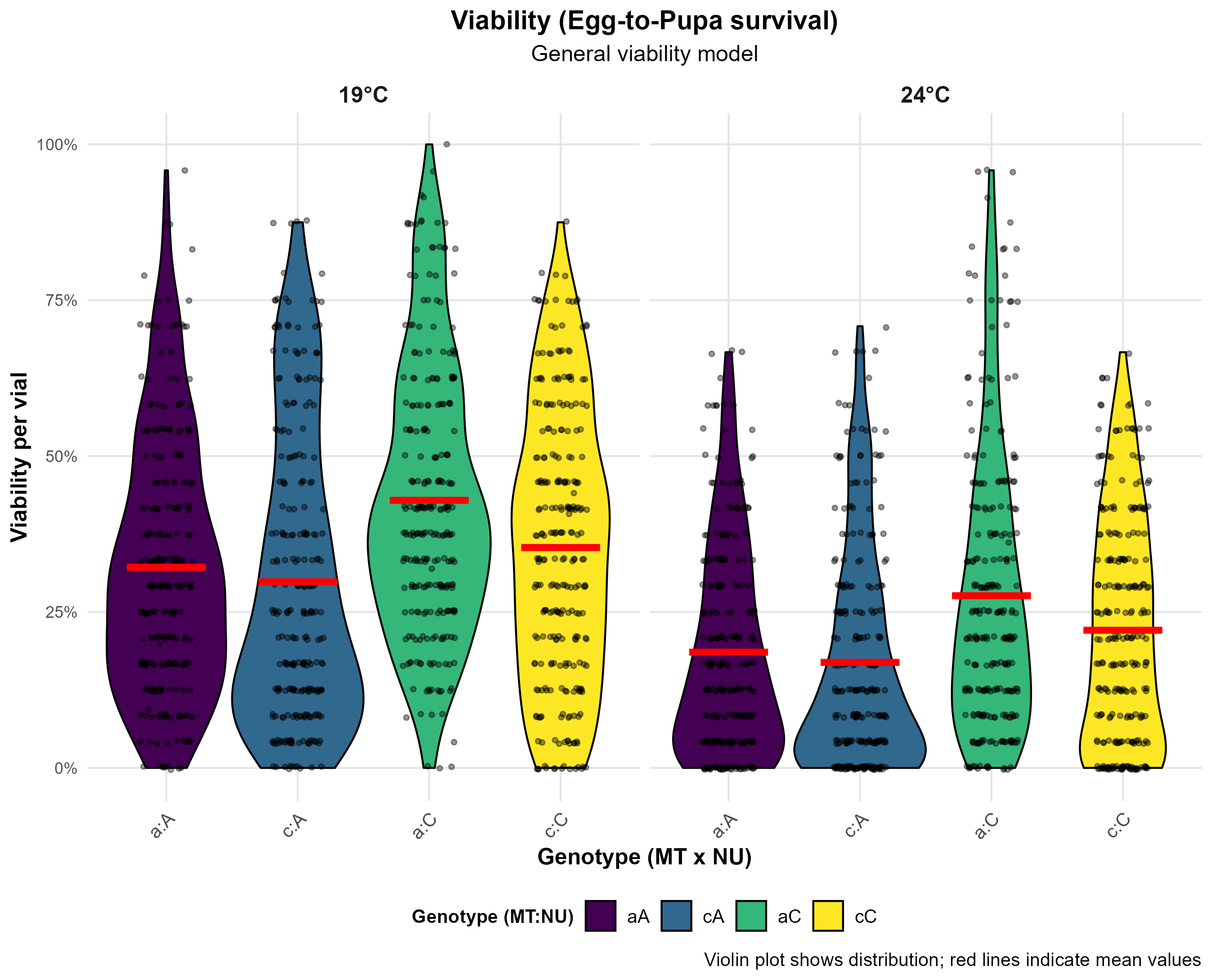

Supplement: Supplementary file 1 [file insects-16-00415-s001.zip › EtP_viability_plot.png]

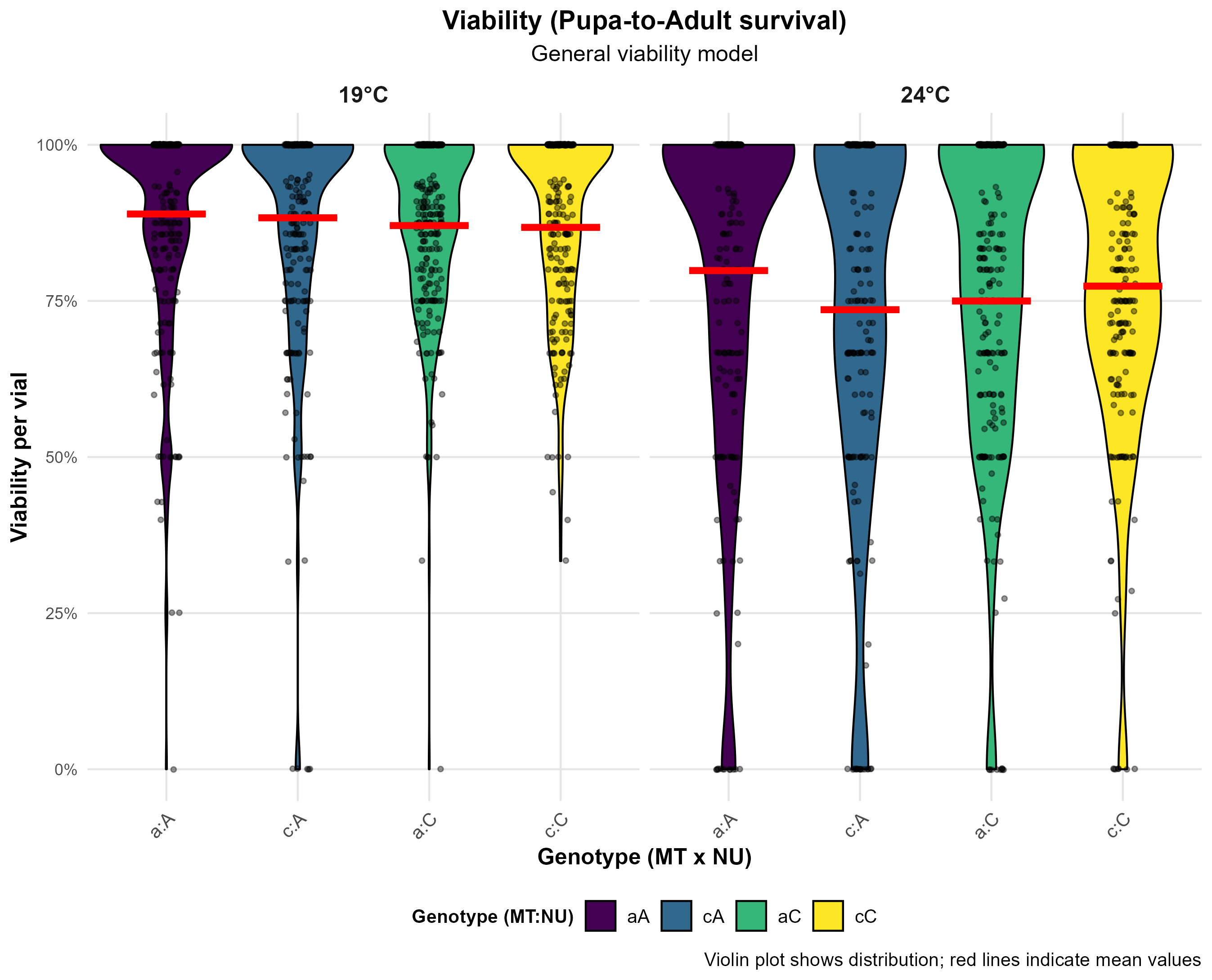

Supplement: Supplementary file 1 [file insects-16-00415-s001.zip › PtA_viability_plot.png]
